# *Candida* in the lung: Fact, fiction, friend or foe?

**DOI:** 10.1371/journal.ppat.1014038

**Published:** 2026-03-10

**Authors:** Philip Mitchelmore, Seána Duggan

**Affiliations:** 1 Academic Department of Respiratory Medicine, Royal Devon University Healthcare NHS Foundation Trust, Exeter, United Kingdom; 2 MRC Centre for Medical Mycology, Department of Biosciences, University of Exeter, Exeter, United Kingdom; University of Maryland, Baltimore, UNITED STATES OF AMERICA

## Introduction

*Candida* species are often dismissed as benign contaminants in respiratory samples. This perception is rooted in historical assumptions of lung sterility and the belief that *Candida* spp. rarely cause pulmonary disease outside severe immunocompromise. As a result, it has shaped diagnostic and treatment practices for decades. However, accumulating evidence challenges this paradigm.

**Fig 1:**
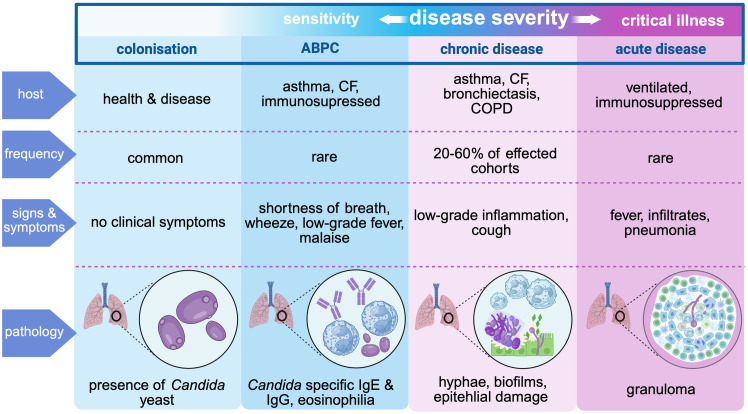
Spectrum of *Candida albicans* involvement in the respiratory tract, from benign colonisation to acute invasive disease. Clinical presentations range from asymptomatic in immunocompetent or ventilated individuals, to hypersensitivity reactions in susceptible hosts (Allergic Bronchopulmonary Candidiasis, ABPC), low-grade inflammation in chronic lung disease, and rare cases of acute invasive disease in critically ill or immunosuppressed patients. The spectrum is stratified by host factors, incidence, and clinical features, illustrating increasing disease severity from left (blue) to right (pink). *CF, cystic fibrosis; COPD, chronic obstructive pulmonary disease.*

Recent studies reveal that *Candida* spp., particularly *Candida albicans*, are not only common in the respiratory tracts of critically ill patients but may also contribute to dysregulated host immune responses, alter bacterial co-pathogen dynamics, and exacerbate lung injury in the critically ill setting. In addition to its role in critically ill patients, such as those who are mechanically ventilated, there is growing recognition that *C. albicans* may act as a pulmonary pathogen in less severe settings. Examples may include during corticosteroid use or when the pulmonary microbiome is disrupted, as seen in COVID-19 and chronic lung disease. Here, we explore the evolving understanding of *Candida* in the human lung, critically examining its role as a coloniser and opportunistic pathogen (Fig 1).

## The respiratory mycobiome

The once-held belief that lungs were sterile was replaced by recognition of the respiratory microbiota, and the understanding that microbial community structure influences health [1]. Early studies focussed on the bacterial components of these communities, and recent years have seen a surge in approaches that broaden insight into the lung mycobiome; however, much of this work has been conducted in the context of specific respiratory diseases. Studies have shown that the healthy lung mycobiome is diverse, with *Candida* spp. often predominating [2]; and *Saccharomyces, Penicillium, Dictyostelium, Fusarium, Aspergillus, Davidiellaceae,* and *Eurotium* also detected [3]. However, composition is altered depending on specific disease state for example, *C. albicans* is typically increased in the mycobiome of people with chronic obstructive pulmonary disease (COPD) and cystic fibrosis (CF), but not in people with asthma [2,4].

An integrative microbiomics study has helped clarify how *Candida* fits within complex airway microbial communities [5]. In bronchiectasis cohorts, airway bacteria, viruses, and fungi were analysed using network-based approaches that prioritised microbial interactions over individual organism abundance. Patients at higher exacerbation risk exhibited less interconnected airway communities, with more negative or competitive relationships between microbes. These disrupted interaction patterns intensified during exacerbations and returned toward baseline following treatment, indicating that shifts in microbial interactions align with clinical state. Within these multi-kingdom networks, *Candida* spp., including *C. albicans*, were among several taxa frequently connected to other community members. This connectivity reflects co-occurrence within the airway microbiota rather than dominance by abundance or evidence of a direct pathogenic role. Importantly, exacerbation risk was more closely linked to patterns of microbial interaction than to the presence or amount of any single organism, indicating that, in the context of *Candida* spp., worsening of airway disease is better explained by community behaviour than by individual microbes.

Together, these observations raise the question of whether *C. albicans* merely coexists within the lung or contributes to disease in specific host contexts.

## Reconsidering *Candida albicans* in respiratory disease

To address this question, we integrate experimental, observational and clinical evidence to examine when, and under what conditions, *C. albicans* may influence respiratory disease.

### Mechanistic insights from experimental and translational studies

Although *C. albicans* is best recognised as a cause of mucosal and bloodstream infections [6], it possesses traits that may be relevant within airway environments. These include adhesion to epithelial surfaces, biofilm formation on mucosa and indwelling devices, and secretion of proteases capable of modifying local immune responses [7]. Other classical virulence attributes, such as morphological plasticity and immune evasion strategies characterised in invasive disease, are not lung-specific but may become relevant under particular airway conditions, including epithelial injury, impaired immune surveillance or prolonged antimicrobial exposure [8]. Together, these observations are consistent with the idea that *C. albicans* behaviour in the lung varies with host context and airway conditions.

Experimental models provide insight into how these traits might influence airway pathology. In in vitro and murine systems designed to reflect cystic fibrosis-like environments, where neutrophil-driven inflammation predominates, *Candida* biofilms can impair neutrophil killing and amplify protease-mediated tissue damage [9]. Conditions relevant to COPD and bronchiectasis, including recurrent epithelial injury, corticosteroid exposure, and repeated antibiotic use, further promote fungal persistence and biofilm formation on compromised airway surfaces [10,11]. In murine models of respiratory exposure, *C. albicans* has been shown to induce lung damage, elevate inflammatory cytokine signalling (e.g., IL-1β, IL-6, IL-17), impair bacterial clearance, and exacerbate epithelial injury [12,13]. However, these findings must be interpreted cautiously, as many models employ high fungal burdens or hypha-promoting conditions that may not accurately reflect stable colonisation scenarios in humans. Indeed, in a contrasting study, short-term *C. albicans* colonisation reduced bacterial burden and lung damage, underscoring the complexity of pathogen–pathogen interactions in the airway [14].

*C. albicans* may be implicated in allergic airway disease via the gut–lung axis. In murine models, antibiotic-induced gut dysbiosis with *C. albicans* overgrowth expands lung-resident ILC2s and enhances Th2-driven airway inflammation. In parallel, human observational data from a small asthma cohort show that higher intestinal fungal-to-bacterial DNA ratios are associated with more severe asthma exacerbations, independent of recent antibiotic or glucocorticoid exposure, supporting the existence of a gut–lung fungal axis in allergic airway disease [15]. In a study examining over 30 fungal species, *C. albicans* emerged as the most potent inducer of IL-17-producing CD4^+^ T cells in human blood. Importantly, some of these T cells recognise conserved epitopes shared with *Aspergillus fumigatus*, raising the possibility of immunological cross-reactivity linking gut colonisation with Th17-dominant lung pathologies, including neutrophilic asthma, bronchiectasis, and COPD [16].

### *Candida albicans* in chronic lung disease

Immune-mediated airway disease provides an example in which *Candida* spp. have been implicated in pulmonary pathology.

Allergic bronchopulmonary aspergillosis (ABPA) is a well-recognised complication of chronic airway disease, and a related but less commonly diagnosed entity, allergic bronchopulmonary candidiasis (ABPC), has been described in association with *Candida* spp. [17]. ABPC is a hypersensitivity disorder resembling ABPA and is characterised by recurrent pulmonary infiltrates, elevated total IgE, and *Candida*-specific IgE. ABPC remains under-recognised due to overlapping clinical features and limited awareness. However, reported cases suggest that, unlike ABPA, bronchiectasis is not a consistent defining feature. Importantly, ABPC typically responds to corticosteroids and antifungal therapy, paralleling treatment strategies used for ABPA [18].

In people with chronic lung diseases, *Candida* spp. colonisation is common and increasingly recognised as clinically relevant. In patients with bronchiectasis, mucus plugging, or collapsed lung, *C. albicans* is commonly isolated from respiratory specimens and associated with persistent airway obstruction, with some reports of clinical improvement following antifungal therapy [19]. In cystic fibrosis (CF), the point prevalence of *Candida* in sputum ranges from approximately 40% to 60% [20], with *C. albicans* predominating, followed by *Candida glabrata (*now *Nakaseomyces glabratus)* and *Candida parapsilosis* [19]. Longitudinal data further suggest species-specific effects; in a 16-year retrospective study, carriage of *Candida dubliniensis* was associated with accelerated decline in FEV_1_ [20]. In non-CF bronchiectasis, reported *Candida* carriage is approximately 30%, with *C. albicans* associated with exacerbations [21]. In COPD, between 10% and 40% of patients are colonised during stable disease, with prevalence exceeding 90% during acute exacerbations [22]. This marked increase during exacerbations supports the idea that fungal dysbiosis, rather than simple presence, accompanies periods of disease instability.

Within diseased airways, *C. albicans* frequently co-colonises alongside bacterial pathogens as part of polymicrobial communities [2,11,23]. Inter-kingdom interactions shape community dynamics—for example, *C. albicans* can form robust biofilms with bacteria like *Pseudomonas aeruginosa*, exchanging chemical signals that promote mutual persistence: fungal ethanol production stimulates bacterial cyclic-di-GMP signalling and biofilm formation, while *P. aeruginosa*-derived phenazines enhance fungal ethanol production, creating a positive feedback loop that may reinforce chronic co-infection [24]. Ultimately, these microbial interactions are associated with worse clinical outcomes: for instance, bronchiectasis patients often harbour *C. albicans* (and other fungi such as *Aspergillus fumigatus*) alongside *P. aeruginosa*; this co-presence has been linked to persistent airway inflammation and microbiota imbalance [21]. Such polymicrobial biofilms are associated with increased tolerance to inhaled antibiotics in CF and bronchiectasis, particularly in communities involving *P. aeruginosa* and *Staphylococcus aureus*, within which *C. albicans* commonly coexists [25]. In addition, *C. albicans* secretes proteases that degrade the host antimicrobial peptide LL-37 into truncated, inactive forms, potentially impairing microbial clearance and favouring persistent, exacerbation-prone communities [26]. Collectively, these findings position *C. albicans* as a participant within complex airway ecosystems capable of influencing disease trajectories through interkingdom interactions, while leaving open the question of causality.

### *C. albicans* in immune-modified and critical illness contexts

During critical illness *C. albicans* colonisation of the respiratory tract is frequent, particularly among mechanically ventilated patients, and may hold prognostic significance [27]. A systematic review and meta-analysis of ventilated ICU patients have linked *Candida* airway colonisation to prolonged mechanical ventilation, increased mortality, and a higher incidence of bacterial co-pathogens, including *P. aeruginosa* and *Acinetobacter baumannii* [28]. These studies underscore the need for well-designed prospective studies to clarify clinical significance.

The COVID-19 pandemic further highlighted the complexity of pulmonary *Candida* in critical illness. Several studies identified *C. albicans* as the dominant fungal coloniser in the lungs of patients with COVID-19, coinciding with fungal dysbiosis and microbiome disruption [29–31]. Impaired host immune responses to *C. albicans*, but not *A. fumigatus*, have also been reported in this setting, suggesting pathogen-specific immune dysfunction [32]. Critically ill COVID-19 patients also experienced higher rates of invasive candidiasis than non-COVID ICU patients [31], reinforcing the importance of vigilance in *Candida* surveillance. Notably, azole treatment was associated with increased abundance of *Candida* spp. and a corresponding reduction in *A. fumigatus* within the lung mycobiome [29]. Together, these observations suggest that *C. albicans* may act as a marker or modifier of disease severity in critical illness, although definitive evidence of causality remains limited.

## Diagnostic and therapeutic considerations

The diagnosis of pulmonary candidiasis remains inherently difficult. In one illustrative case, histopathological confirmation of *C. glabrata* in a borderline immunocompetent host required two separate lung biopsies before fungi were identified [33]. This underscores the limitations of current diagnostic tools and the difficulty in distinguishing colonisation from true infection, particularly in patients without classic immunosuppression. In low-biomass environments such as COPD and mechanically ventilated lungs, upper-airway contamination can confound sample interpretation. Techniques such as protected brush sampling or BAL, combined with fungal PCR or next-generation sequencing, may improve diagnostic specificity. Measurement of (1,3)-β-D-glucan in BAL fluid has also been explored as a potential adjunct marker of fungal burden, although its specificity for *Candida* and clinical utility in this context remain uncertain [34].

Current guidelines advise against antifungal treatment for *Candida spp.* detected in respiratory specimens in the absence of invasive disease, particularly in immunocompetent individuals. This reflects the prevailing view that pulmonary *Candida* colonisation is not clinically significant. Even when therapy is indicated, there are complications: In patients on long-term azole prophylaxis, a shift toward non-*albicans* species is often observed, alongside the emergence of fluconazole-resistant isolates [35]. Although some studies suggest that antifungal therapies such as nebulised amphotericin B or itraconazole mouthwash can reduce *Candida* burden and mucus viscosity in CF [36], systemic azoles are limited in cystic fibrosis due to toxicity and interactions with *CFTR* modulators [37]. Furthermore, the CANTREAT trial found no clear benefit of antifungal therapy in ventilated patients colonised with *Candida* [38]. A recent systematic review and meta-analysis of 3,802 patients similarly found no impact on mortality, mechanical ventilation duration, ICU stay, or secondary infection rates [39]. These findings reinforce recommendations against routine antifungal therapy for *Candida* decolonisation without confirmed invasive disease.

In summary, although *Candida* colonisation may have pathological and ecological consequences, current evidence does not justify antifungal treatment based on detection alone. The field would benefit from improved diagnostic tools and large, prospective trials to define when, if ever, antifungal therapy is warranted—particularly in the context of critical illness or chronic lung disease.

## Outlook

While *C. albicans* is well recognised in both clinical and research contexts, its role in respiratory disease remains poorly understood. It is now clear that *Candida spp.* can be components of the respiratory microbiota and that alterations in their abundance are associated with a range of respiratory diseases. But can *Candida spp*. themselves cause respiratory disease? The answer is both straightforward and nuanced. On one hand, case reports throughout the literature clearly document instances of *Candida spp.* causing respiratory disease, [ 17,18,40–46]. On the other hand, classic respiratory infections such as pneumonia are so rarely attributed to *Candida* that scepticism about its clinical relevance in the lungs justifiably persists.

We do not seek to divert attention from understudied pulmonary fungal pathogens such as *Pneumocystis jirovecii* and *Histoplasma capsulatum*, which are responsible for severe and often life-threatening infections. However, the evidence presented here positions *C. albicans* as a potentially underappreciated yet significant contributor to lung pathology—one that merits substantially more investigation alongside other fungal threats. A major barrier to progress is the lack of reliable diagnostics for *Candida* respiratory infections; overcoming this challenge will require both the development of improved diagnostic tools and increased clinical awareness.
